# Family Caregivers’ Experiences of Formal Peer Support in a Finnish Setting – a Socioecological Understanding

**DOI:** 10.5334/ijic.9104

**Published:** 2025-07-24

**Authors:** Sarah Åkerman, Fredrica Nyqvist, Annika Wentjärvi, Laura Coll-Planas

**Affiliations:** 1 Social Policy, Faculty of Education and Welfare Studies, Åbo Akademi University, Vaasa, Finland; 2 Social Policy, Faculty of Education and Welfare Studies, Åbo Akademi, Vaasa, Finland; 3 Health and Welfare, Novia University of Applied Sciences, Vaasa, Finland; 4 Research Group On Methodology, Methods, Models and Outcomes of Health and Social Sciences [M3O], Faculty of Health Sciences and Welfare, Centre for Health and Social Care Research [CESS], University of Vic-Central University of Catalonia [UVic-UCC], Vic, Spain; 5 Institute for Research and Innovation, Life Sciences and Health in Central Catalonia [IRIS-CC], Vic, Spain

**Keywords:** peer support, ageing, informal care, voluntary, community, third sector, Finland, welfare

## Abstract

**Introduction:**

Peer support programmes are important complementary support forms in integrated care. This study investigates formal peer support experiences among family caregivers in Finland by scrutinising multidimensional input and output mechanisms of emotional, informational, and appraisal support.

**Methods:**

Seventeen family caregivers were interviewed through focus group and individual interviews. The qualitative data was analysed using a socioecological model for formal peer support.

**Results:**

The family caregivers described their caregiving situation as a source of pain enabling receiving emotional support from peer support participants with similar experiences – although norms of privacy and taboo hindered participation. Organisational aspects of programme delivery such as setting expectations, rules of confidentiality, environmental setting, group dynamics and leadership influenced experiences of informational and appraisal support. Overburden created barriers to receiving emotional and informational peer support. The participants wanted to influence society and contribute to other family caregivers through formal peer support – highlighting its socioecological nature.

**Conclusions:**

Each attribute of peer support [emotional, informational, and appraisal support] formed its own, yet interlinked, ecosystem. Experiences of peer support are beyond programme delivery, and future studies could employ a socioecological framework when further delving into attributes of formal peer support among family caregivers in specific target groups and/or settings.

## Introduction

In search of sustainable care solutions for the ageing population, family care has been raised on the political agenda across the world [1,2]. Family care refers here to either paid or unpaid care provided by friends and family members with a previous relationship to the care recipient. While family caregiving can be linked to positive experiences and health advantages for the caregiver [2,3], it may also entail emotional, practical, and psychological stressors with a negative impact on the caregivers’ health, economy, and social life [4]. Public support forms such as respite care and economic allowances have been developed in many welfare states [5]. However, family caregivers may still struggle to utilise support due to issues with information seeking, norms of obligation towards the care recipient, worries about the quality of services, or difficulties with needs assessment [6,7]. Formal peer support activities, typically organised and delivered by the voluntary and community sector (VCS), are therefore designed to mitigate some of these challenges by offering caregivers emotional, informational, and appraisal support from those with similar experiences [8,9]. Emotional support refers to expressions of care, reassurance, attentive listening, and encouragement, while informational support refers to providing knowledge relevant to problem-solving [8]. Appraisal support affirms the appropriateness of emotions, cognitions, and behaviours pertinent to self-evaluation, reassurance and motivation [8]. The present study will investigate the multidimensional mechanisms influencing experiences of formal peer support among family caregivers in the context of Finland from a socioecological perspective [10]. Thus far, there is limited knowledge on the socioecological nature of peer support in the context of family care. A socioecological model emphasizes the interplay between individual, relational, community, and broader systemic mechanisms in shaping health and well-being [10,11]. A socioecological perspective includes analysing multilevel input and output perspectives where participants are seen not only as recipients of a given programme content, but rather active participants themselves transforming society through mobilisation and active participation in peer support programmes [10].

### Previous research on peer support and family care

Peer support programmes are generally seen as important complementary support forms for family caregivers [12,13]. A previous scoping review on peer support for caregivers of someone with dementia confirmed that peer support interventions hold the potential to improve health and wellbeing of informal caregivers, although the effectiveness may not always be possible to prove statistically [13,14]. The wide range in programmes and outcome measures also challenges the ability to draw any universal conclusions on the effects of peer support programmes for family caregivers [13,14]. In general, caregiving is believed to cause a wide range of stressors that may entail for example role conflicts, stress, and isolation [4,15]. This underlines the role of support and the function of “being there” which is at the core of peer support [10]. Previous studies exploring experiences of support needs have found that family caregivers describe complex care needs, uncertainty and lack of information [7,16,17] highlighting needs of informational support provided at the right timing and under the right circumstances. In this sense, peer support provides a forum where information can be provided spontaneously and with a low threshold. Similarly, appraisal support is important to enable and strengthen family caregivers’ awareness of their rights and position as a family caregiver. Interventions focusing on peer support for family caregivers tend to assess psychosocial outcomes and outline programme delivery [13,14], leaving out broader societal structures and the specific attributes of emotional, informational, and appraisal support embedded in peer support [8,9,10]. The present paper therefore makes a distinct contribution to the research field on peer support and family care by analysing experiences of peer support from a socioecological perspective in a Finnish setting.

### Carers in Finland

In Finland, family caregivers can be supported through the public Informal Care Allowance (ICA) which is a social service package for the care recipient, where some of the benefits are directed to the caregiver [18]. The ICA includes an economic allowance, monthly respite care, pension credits, accident insurance, other needed social and health care services for the care recipient, and the right to training and health examinations according to need for the caregiver [ibid]. Out of an estimated 350 000 family caregivers in Finland [19], about 50 000 receive the ICA [20]. About 67% of ICA family caregivers are aged 65 and most of them provide intensive care to a co-residing spouse with cognitive decline [21] which is known to be one of the most burdensome care contexts [4,22]. In Finland, non-governmental organizations (NGOs) are important sources of social support for family caregivers [23,24]. Family caregivers’, and older care recipients’, social needs are often left unmet in public social and health care increasingly prioritizing medical needs [23,25,26]. Public funding for NGOs for organizing social activities, including formal peer support, is partly available through competitive funding schemes [24,27].

In Finland, formal peer support activities targeted towards older adults and family caregivers are led either by professionals or by lay people themselves, typically pensioneers. Indeed, to achieve cost efficacy, work tasks among NGO staff members increasingly include coordination and education of lay people to carry out activities. Additionally, engaging older adults in volunteering tasks is increasingly part of global and national health policies emphasising healthy ageing [2,28,29]. Consequently, formal peer support activities for family caregivers in Finland are led either by professionals, and/or increasingly by lay people who have completed a training course for their assignment [30]. While formal peer support can include instrumental support referring to practical support aiming to reduce the care load [10], the main aim of formal peer support in a Finnish context is to support family caregivers through preventive emotional, informational, and appraisal support.

The socioecological model emphasizes the input and output perspectives included in the interplay between individual, relational, community, and broader systemic mechanisms [10,11]. In the context of family caregiving in Finland, this model is particularly relevant as it highlights how caregiving experiences are not only influenced by individual mechanisms (e.g., health, coping abilities) on the micro-level but also by the environment, including the availability of support services at the meso-level, societal norms, and the policy context at the macro-level. In Finland, where the welfare system is undergoing significant changes in terms of austerity measures and centralisation of social and health care services, the role of peer support in meeting the emotional, informational and appraisal needs of family caregivers become even more significant. Additionally, a socioecological framework [10] allows for analysing the bidirectional directions of peer support programmes, where the participants also influence society through the outcomes of peer support programmes. This study aims to apply a socioecological perspective to understand peer support dynamics among family caregivers, particularly older spousal caregivers, in a Finnish setting.

## Aim and research questions

This study aims to investigate Finnish family caregivers’ experiences of formal peer support from a socioecological perspective. The research questions are:

a) How do older family caregivers experience different attributes of formal peer support such as emotional, informational, and appraisal support?
b) What kind of micro-, meso-, and macro-level mechanisms influence family caregivers’ experiences of emotional, informational, and appraisal support through formal peer support?

## Data and method

The data is based on interviews with 17 family caregivers collected between October 2023 and January 2024. The respondents were contacted via a contact person working at an NGO providing formal peer support activities for family caregivers in Western Finland. The NGO, which will not be named in the study for anonymisation reasons, provides training for volunteers with personal experience of family care to prepare them for offering individual and/or group-based formal peer support activities either physically, via telephone, or virtually for current family caregivers. The data was collected through four qualitative focus groups (three to four participants per group) and two individual interviews. All interviews, except for one, were conducted onsite either in the participants’ homes or in meeting rooms at the NGO. One individual interview was conducted via telephone. The focus group as well as individual interviews lasted between 45 and 65 minutes. The interviews were based on semi-structured interview guides that revolved around themes such as changes in one’s social network due to caregiving, the experiences of formal peer support activities, and the role of VCS in support for family caregivers. All interviews were recorded and transcribed. Reflective notes were taken by the researcher(s) immediately after the interview(s).

The contact person at the NGO used a purposeful sampling method to identify family caregivers with rich experience of participating in formal peer support activities. The contact person at the NGO personally informed potential participants about the research project and gave the contact information to the main responsible researcher (SÅ). SÅ contacted the potential respondents via telephone and agreed on dates for the interviews. At the data collection occasion, the participants received written informed consent and background information forms to complete. In the case of individual interviews, templates for written informed consent and background information were posted to the respondents’ homes and signed and/or filled in documents were returned via post to the researcher prior to the interview. Descriptive statistics of the family caregivers are presented in Table 1.

**Table 1:** Descriptive characteristics of the study population (n = 17).


Sex	

Female	13

Male	4

Mean age of carer	76.2

Mean age of care recipient	78.7

Marital status	

Partner	16

Single	1

Co-habiting	13

Yes	4

No	

Type of residence	

Urban	12

Rural	5

Educational level	

10 years or more	15

Less than 10 years	2

Relationship to care recipient	

Spouse	16

Parent	1

Main reason for providing family care	

Cognitive decline	12

Physical	9

Other	1

Years (mean) of providing family care	8.4

Receives Informal Care Allowance	10

Experience of individual formal peer support	5

Experience of group-based formal peer support	14


Ethical approval was deemed not necessary for this non-medical research project according to criteria set by the Board for Research Ethics at Åbo Akademi University and the guidelines of the Finnish Ethics Committee for the Human Sciences [30]. Written consent was gathered from all participants in advance of interviews and confirmed verbally at the time of interviews.

### Theoretical and methodological framework: socioecological model

**Figure 1:**
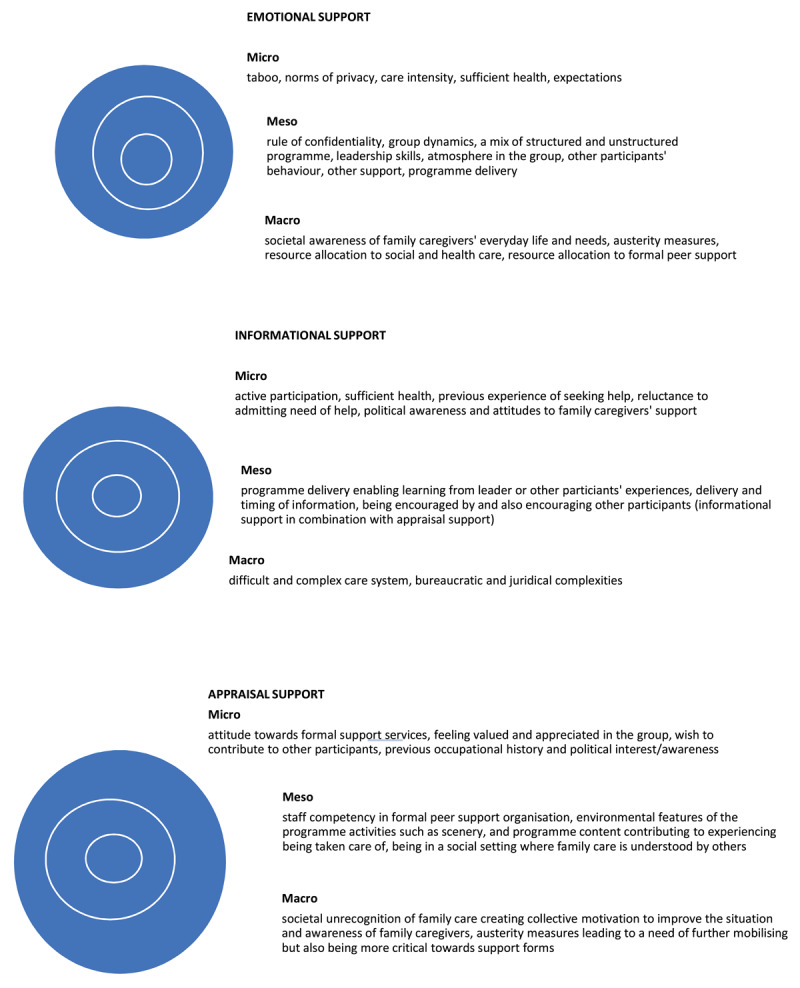
An illustrative summary of mechanisms influencing family caregivers’ experiences of emotional support, informational support, and appraisal support from a socioecological perspective.

The present study uses a socioecological model of peer support [10] as a theoretical framework for the analyses. The analyses were conducted using qualitative content analysis with a directed approach [31]. According to Evans [10], the theoretical underpinnings of peer support are multidisciplinary with health-promoting effects of social support being the main driver. Peer support underscores the value of personal knowledge about illness [or in the present study, also family care]. Additionally, Evans [10] includes stigma in her model, which adds an important social and cultural context for understanding care, illness and the inputs and outputs of peer support programmes that goes beyond programme delivery. In the analyses of the present study, formal peer support is not only analysed through its input and outputs on micro, meso, and macro levels, but also as scrutinised into different attributes of peer support. The data was first categorised into overarching themes of experiences and influencing mechanisms of emotional, informational, and appraisal support [8,9,10]. The three overarching themes were then deductively divided into subcategories of micro, meso, and macro level mechanisms [10,11]. Micro level mechanisms include for example experienced norms, attitudes and care load. Meso level mechanisms refer to the private social network, and organisational and intrapersonal aspects of the formal peer support activities. The macro level refers to political and societal structures on a national, regional, or municipal level. As a further step in the analyses, the results and categories were reread to look for input versus output mechanisms [10]. These mechanisms were not defined separately, but instead included in the different micro, meso, and macro levels and discussed in the results section. When going through the data, the researchers inductively identified a fourth theme of “not feeling supported” which was added with the same subcategories as the original three themes. When the first analyses including the four themes were conducted (presented in Results), a secondary analysis was done to summarise the mechanisms influencing experiences of emotional, informational, and appraisal support through formal peer support (Figure 1). In Figure 1, the theme of “Not feeling supported” was merged into the other three themes.

## Results

The results are summarised in Figure 1 at the end of the Results.

### 1. Emotional support – experiences and influencing mechanisms

When someone in the family gets sick, everything changes.

*Female family caregiver of husband and child*

Most of the family caregivers described major changes in their social network due to the caregiving situation, although the exact consequences differed. Some participants received more emotional support from their private social network, while others experienced a greater need to rely on new contacts facilitated by formal peer support activities. The needs of emotional support also varied depending on the health and prospects of the care recipient. One family caregiver described caregiving as a process of grieving, which motivated the needs of emotional support from someone who had experienced a similar process.

/…/ it is a process of grieving. In different ways. You notice that your close one is getting worse and worse. So it’s good to get to discuss with others who have went through the same thing.

*Female spousal caregiver*

Receiving emotional support in formal peer support activities was described by one participant as the ability to show up authentically and burst into tears without having to disguise the negative feelings.

It is a new life situation, but going to those meetings, I was so tired when I got there, so sometimes I just sat there and cried when I told them about my situation, because that was the only thing I managed to do.

*Female spousal caregiver*

Several family caregivers described receiving emotional support by discussing their situation. One family caregiver described “pouring out” her burdens in a formal peer support group meeting, while she noted that the other participants mostly kept quiet. This family caregiver seemed to experience less stigma associated to the circumstances of everyday life and the care recipient’s health status than some of the other family caregivers.

When we meet in a formal peer support group, the others usually say their name and who they care for and not really anything else. And I just pour everything out to get support through sharing, it’s not the only place where I talk about it.

*Female spousal caregiver*

Other family caregivers described difficulties with sharing personal information about the care recipient leading to feelings of being unloyal towards their care recipient, usually their spouse. Feelings of pride in terms of not wanting to admit a need for help, and feelings of shame related to the care recipient’s health were mentioned as hindering participating and receiving emotional support. The family caregivers emphasised the importance of organisational arrangements. Being able to talk about sensitive topics in the formal peer support activities were facilitated by a rule of confidentiality in the group, and by the group members sharing similar characteristics regarding the care context. In this way, individual norms and values at the micro level hindered the ability to participate and receive emotional support in the formal peer support group, while on a meso level, organisational aspects facilitated participation.

I have been going to an association for dementia and it has been very good. There we get to gather and talk. It is a rule of confidentiality of course. Because it is not so easy to tell difficult things about close ones.

*Female spousal caregiver*

Micro and meso levels were intertwined in one family caregiver’s experiences where a one-on-one formal peer support relationship was facilitated by organisational aspects. The family caregiver attended a meeting where the volunteer presented, which lowered the threshold for the family caregiver to contact the volunteer later. The peer support relationship had been functioning smoothly without specific rules or agreements for when and how to keep in contact. The family caregiver appreciated the flexible arrangements allowing for keeping in contact when the timing was right, rather than participating in formal peer support group activities at a specific time and place. This flexibility was however insufficient at times when the care burden was extensive, but at the time of the data collection, it was easier to keep in contact because the care recipient no longer lived at home.

Sometimes she [volunteer] calls, sometimes I call. I think it works well. Everything else is so scheduled, but we get to meet when we can make it work, as simple as that. We have met in different ways, sometimes I went to her house, she can come to my house now that I am alone. It didn’t work before that.

*Female caregiver with experience of providing care to her husband and child*

Indeed, the family caregivers’ ability to engage in formal peer support activities were highly dependent on the health of the care recipient and/or opportunities to arrange respite care through formal services or the private social network. One family caregiver mentioned that when the needs of emotional support are the most extensive, the family caregiver may simultaneously be too exhausted and preoccupied to engage in support activities.

### 2. Informational support – experiences and influencing mechanisms

I was so lost at first when I became a family caregiver.

*Female spousal caregiver*

Several family caregivers described receiving important information about economic, juridical and instrumental support by listening to the experiences of other family caregivers at formal peer support meetings. Even at the time of the data collection, advice was shared among the participants during the focus group interviews. The family caregivers described the public social and health care system as complex and often a source of frustration, despite simultaneously experiencing that they were met with kindness by individual staff members. The family caregivers also described excessive administrative burden associated to family caregiving in terms of tax reductions, user fees, bureaucracy, and other juridical matters. Utilising the information provided in the peer support activities was further facilitated by a combination of informational and appraisal support, where the other attendants personally encouraged each other to seek the services and benefits.

One family caregiver especially stressed the need for group leaders in peer support activities to be able to arrange meetings that provide relevant information. This participant stressed that, due to his care situation differing from the other care recipients’, information and advice were the most effective forms of support attainable through the formal peer support group. According to him, he could not receive emotional support from the other participants due to a lack of understanding resulting from different care contexts.

### 3. Appraisal support – experiences and influencing mechanisms

One participant described a structured peer support occasion in which family caregivers received foot massage together. This experience was enabled by comparatively resourceful arrangements organised by an NGO and resulted in a shared experience of being valued and recognised – both as individuals and as a group representing family caregivers.

I was just thinking about a camp [for family caregivers] I visited, it was such a shared pleasure when we were there last time and had a foot bath and massage together. /…/ it was the feeling of security and belonging that was really nice. And to think that now somebody is taking care of you, that it is not the other way around where we take care of someone else. That feeling was very nice.

*Female spousal caregiver*

Several family caregivers also mentioned a camp for family caregivers that had taken place by the sea. The environmental features further added to the feeling of being appreciated and valued both individually and collectively.

Appraisal support was sometimes obtained by listening to other family caregivers whose circumstances were more challenging than one’s own. Perspective was also gained by being heard and recognised by the other participants – and by being made aware of one’s efforts and entitlement to public support. Being able to share about one’s burdens was necessary to receive appraisal support, which, just as in the case of emotional support, was challenging for some family caregivers.

It’s a very intimate thing to come and say, to admit that you are not managing. /…/ But you have to admit it, because you are not going to manage forever. No matter how much you love your husband. Sometimes I just want to lay down and cry. But now I try to improve this, our life, by accepting all the help I can get. And try to organise everything.

*Female spousal caregiver*

In the example above, one family caregiver described the process from initially feeling reluctant towards accepting help to realizing that it was a way of making the family caregiving situation more durable and sustainable. Being encouraged to seek help was described as important form of appraisal support through formal peer support, which could further transcend into acquiring instrumental support from other sources.

The participants also described reciprocal actions taken by themselves in formal peer support meetings. They wished to contribute to other family caregivers’ well-being and awareness of their rights, by encouraging them to seek and/or accept support services. Contributing to others and spreading awareness seemed to be especially relevant for participants who had additional (informal) peer supporters in their private social network, and/or for participants with high levels of political awareness and engagement. One participant expressed a wish to contribute to greater recognition of family caregivers’ situation by participating in the data collection.

It would be important to get the information out there how important this is, and how many we are who do this, we as family members. We do our job. And how much are we saving to society? I would like that to get out there, because few people understand this. We who are family caregivers save millions of euros to society.

*Female spousal caregiver*

### 4. Not feeling supported – experiences and influencing mechanisms

I don’t think that I have received any support, and I don’t know in which shape that support should come.

*Male spousal caregiver*

While all family caregivers described at least one experience of receiving either emotional, informational, or appraisal support through formal peer support activities, several family caregivers simultaneously described an experience of not being supported. This also emerged in the quote above where a male caregiver expressed that he had not received peer support and was uncertain how such support should be provided.

One family caregiver mentioned that getting adequate medical care for his wife would be the best alleviator for him, which reflected hopes a hope for either instrumental support or a transformed life situation. In this sense, the caregiver felt unsupported although his expectations could not have been met through peer support in the first place. His need for additional help still influenced his perception of peer support programmes.

I have not received any support. The best alleviator for me would be if my wife would get help.

*Male spousal caregiver*

A few other family caregivers also expressed hopes that formal peer support programmes would provide respite care, which illustrates risks of false expectations of the programmes.

Other criticism related to the content and focus of the group-based formal peer support activities. One participant expressed disappointment with a meeting that focused on a recently widowed participant, which was an irrelevant context awakening negative emotions in him.

It was explained to me that in formal peer support groups, we are supposed to support each other. And it’s a good thought, but it doesn’t really work in my opinion. Because it is not structured in a way so that you can do that. You do listen to each other, but one time we had a meeting where I only became in a bad mood after attending it. One had recently become widowed and naturally experienced a need to talk about that, and did that. But my wife is not so ill that I expect her to die anytime soon. And I thought to myself, what are you guys in the group thinking now, we get to listen to a sad story which we will all recognise ourselves in, eventually. And it felt like this: this is not really support to listen to people who are sad.

*Male spousal caregiver*

Other participants described issues with the behaviours of formal peer support group members, which contributed to a feeling of not being supported. Passive behaviour of other participants led to feelings of frustration.

There was a lot of whining, and they say, “if we only get the social worker here so we can ask her questions”. When they visited us, only one was prepared with questions, and all the rest just sit there and say nothing.

Female spousal caregiver

Issues with badmouthing in the formal peer support group meetings were also described as contributing to the experience of not being supported. One participant described feeling angry and disappointed with herself after attending a meeting where the other members of the formal peer support group criticised local health care staff.

It is a lot of bad talk. One time I was so angry when I got home that I told my husband “I am not going there again”. At night, I was angry at myself for not standing up for myself. That they whine on the local healthcare ward, that they do nothing. I think that they [the staff] run all the time and that we get treated so well by them.

*Female spousal caregiver*

Among a few family caregivers, criticism was raised towards the formal peer support group leaders’ ability to lead group conversations in a constructive manner. The criticism was raised mainly by those with a history of leading group themselves in their occupational history and/or by those who seemed to be overburdened. Simultaneously, in most cases, the criticism was hypothetical rather than based on actual experiences. When discussing how to organise well-functioning group, the family caregivers emphasised the importance of every group member understanding the purpose of the meeting. Relevant material could be used by the group leader to provide the participants with something comforting or positive to think about. In contrast, another participant described such elements as “artificial breathing” and not truly comforting. These examples highlight the diversity of formal peer support programme participants and their preferences.

Several participants stressed the importance of being placed in a group with similar participants. For example, if the care recipients’ diagnosis differed vastly, a feeling of not having anything in common was described by some participants. This was especially evident for a family caregiver who was caring for her child, whose situation differed a lot from the life situation and challenges described by older spousal caregivers.

## Discussion

This study investigated experiences and influencing mechanisms of emotional, informational, and appraisal support through formal peer support among family caregivers in Finland from a socioecological perspective [10]. The results highlight that different attributes of peer support are distinct yet interlinked. The results also highlight the socioecological nature where family caregivers are not only passive recipients of formal peer support programme content, but also active agents aiming to contribute to others and even influence broader society through the programme activities.

Indeed, peer support programmes can be important platforms for societal change and for reducing stigma. The described emotional needs and experiences of formal support could be seen as reflecting contemporary Western society where care and ailment are seen as abnormal phenomena linked to taboo and stigma [32,33]. This social and cultural understanding of the position of care and illness highlights the needs of emotional support offered by an individual or group of people sharing the same circumstances and/or experiences. Simultaneously, experiences of taboo and stigma were not heterogeneously experienced by the participants. One female caregiver did not experience any shame relating to her husband’s dementia, while a male caregiver avoided social events and potential embarrassing moments due to his wife’s cognitive decline. In the latter case, the ”social death” of the person with dementia [33] thus seemed to transfer to the caregiver. Similarly, a female caregiver with a long history of caregiving, first to her child and then later to her husband, described decades of emotional needs met through either close family members or peer support programmes – rather than by the broader community. While the results thus stress the importance of formal peer support programmes for family caregivers, these experiences can also be seen as highlighting a need for further diminishing stigma relating to care and illness in broader society. Future research could especially investigate the facilitating mechanisms among participants who did not feel an urge to isolate from the broader community.

Theoretically, peer support is based on psychological experiences of shared pain [10]. While psychological experiences could be seen as beyond structures and programme delivery, the experiences were was simultaneously deeply interlinked with organisational aspects and broader societal structures – adding support for the socioecological framework suggested by Evans [10]. Indeed, the World Health Organisation [12] also recently concluded that support programmes for family caregivers cannot be viewed in isolation from, but rather in relation to, broader structures and social policies. According to the family caregivers in the present study, insufficient support from other social and health care services increased the need of peer support. Simultaneously, overburden also led to intrapersonal and practical barriers to accessing peer support. When overwhelmed, it became more difficult to attend and actively engage in peer support activities. One participant described only having sufficient energy for crying at a peer support meeting when overwhelmed. Simultaneously, crying and authentically expressing one’s emotions were described as an important form of emotional support facilitated by the formal peer support activity. Apart from a balance in care obligations and resources, the family caregivers also described having to overcome individual norms and attitudes of taboo and stigma to be able to share sensitive information about themselves and their next of kin in the formal peer support activities. Organisational aspects on the meso-level, such as creating groups of participants with similar caregiving characteristics, and applying rules of confidentiality, facilitated engagement in formal peer support activities.

The results showed that experiences of emotional and appraisal support could also be hindered by programme delivery in terms of group dynamics and behaviours of other participants. Potential adverse experiences and perceptions of group-based social support forms have also been noted in other studies [34]. In the present study, a negative atmosphere and participants criticising local service gatekeepers were described as contributing to an experience of not being supported. These results highlight the need of the formal peer support leaders to steer the discussion in a constructive direction and establish rules for suitable discussion topics and ways of conducting. Other criticism regarding programme delivery related to the focus of the content, with two family caregivers expressing that the focus on end-of-life care or death was not relevant for their current situation and caused negative emotions. These examples suggest that formal peer support activities might benefit from clear descriptions of the target group and content to prepare and attract suitable participants. As increasingly pressured social and health care systems might lead to additional involvement of lay-people in formal peer support activities, these results provide important information about the potential negative consequences for participants when the discussions in the meetings are not adequately structured.

According to Evans [10], participants in formal peer support may at first request instrumental and informational support rather than emotional support, although the two former support forms may also include, or lead to, emotional support as well. Receiving informational support through formal peer support was commonly reported by the family caregivers in the present study, and for some, it was deemed the most important support form attainable. Caring for older adults with complex care needs – which is a common care context for older spousal caregivers – may indeed require a lot of information seeking, administration and bureaucracy [6,7]. Additionally, care utilisation among caregivers may be influenced by attitudes, norms, and feelings of obligations [6,7]. Regional variation in support and services may lead to uncertainty about available support forms contributing to further needs of encouragement and information. Formal peer support activities where experts were invited were deemed as effectively providing support, as they provided information while the attendees encouraged each other to seek services. This resulted in a mix of informational and appraisal support with both input and output mechanisms. A previous study found that older family caregivers in Finland and Sweden were more likely to use public support if they were also members of a social and health care organisation or an organisation for patients and/or family members [35] – tentatively reflecting that informational and/or appraisal support gained in VCS may facilitate instrumental support among burdened caregivers.

From a socioecological perspective [10], formal peer support is subject to both input and output at different levels in society. Formal peer support can result in policy advocacy, transform attitudes and decrease stigma. The family caregivers described a wish to contribute to the situation of others in the formal peer support activities. They also requested greater recognition of the economic contribution of family care in society. Some of the family caregivers were critical to the idea of volunteers offering peer support to family caregivers, although when scrutinised, the criticism seemed to be hypothetical rather than based on actual experiences. The negative attitudes also seemed to stem from shortcomings of the overall situation of family caregivers in social and health care. The results further showed that appraisal support seemed to be facilitated by environmental features such as formal peer support activities set in beautiful settings, indicating that these features contribute to participants feeling valued and supported. Environmental aspects of peer support could therefore be further addressed in future research.

## Strengths and limitations

It is noteworthy that at the time of the data collections, planned cuts in social and health care – including VCS – were heavily under debate in media in Finland. The political climate may have contributed to some participants being more critical towards the situation of family caregivers – including formal peer support programmes – in a manner that may not have reflected their true experiences of peer support programmes. Simultaneously, the heavy burden of family caregivers is well documented in Finland, and the planned cuts could be seen as only worsening a situation already acknowledged as vulnerable.

The descriptive table (Table 1) showed that fifteen out of seventeen of the family caregivers were highly educated. This sample represents a rather selective group of family caregivers and contributes to the results being less representative compared to a more diverse sample. Additionally, the participants were recruited from one NGO only which also reduces the ability to generalise the results. However, the aim of the study is not to propose a universal framework of formal peer support, instead, it seeks to initiate a discussion and contribute to a socioecological understanding of the role of formal peer support in integrated care for family caregivers in a Finnish setting.

Some of the participants struggled to understand what formal peer support was and whether they had received it. The interviews revolved mostly around group-based activities, although formal peer support can also be given individually. For the aim of the present study, the distinction between different modes of formal peer support was less important. Nonetheless, future research could further elaborate on distinct modes of formal peer support from a socioecological perspective.

## Conclusion

This paper explored the role of formal peer support among mainly older spousal caregivers in Finland from a socioecological perspective. Formal peer support provided emotional, informational and appraisal support, while simultaneously, family caregivers described an experience of not being supported. Not feeling supported was influenced both by micro mechanisms such as norms and care characteristics, by meso level organisational mechanisms such as programme delivery and group dynamics, and by the macro level development, where austerity measures in social and health care for older adults were under debate at the time of the data collection. Emotional support seemed to require the most effort on an individual level in terms of overcoming stigma and norms of privacy, while the informational and appraisal support were more connected to organisational mechanisms and macro level mechanisms. The attributes of peer support were also interlinked, as a combination of appraisal and informational support seemed to offer informational support most effectively. The results underline formal peer support as an important source of emotional support for family caregivers in a Western culture focused on independence, although formal peer support also entails shortcomings and experiences of not being supported. Programme delivery in group situations was emphasised as important in terms of setting expectations, leading the conversations constructively, creating homogenous groups in terms of caregiving characteristics, and applying rules of confidentiality. Environmental features in terms of beautiful scenery and caring elements in formal peer support programmes were also mentioned as enabling appraisal support.
